# A new program for SELEX sorting and visualization of aptamers

**DOI:** 10.1016/j.omtn.2024.102398

**Published:** 2024-12-12

**Authors:** John J. Rossi

**Affiliations:** 1 Center for RNA Biology and Therapeutics Beckman Research Institute of the City of Hope, Duarte, CA, USA

A recent *Molecular Therapy Nucleic Acids* publication describes a new and improve bioinformatic tool for aptamer sorting and identification from a large pool of possible sequences.^1^ Nucleic acid aptamers are single-stranded RNA and DNA polymers that fold into three-dimensional shapes, which facilitate their binding with high affinity to selected ligands.^2^ Aptamers sprung onto the molecular biology scene following the publication by Turek and Gold of a facile *in vitro* evolution technique termed SELEX (systematic evolution of ligands by exponential enrichment).^3^ This process involves creating a random library of tens of millions of sequences, followed by challenging the library with the target protein or molecule of choice and then capturing the target and bound nucleic acids and counter selecting to eliminate nucleic acids with non-specific target affinity.

The sequences that bind to the desired target are copied and amplified by the polymerase chain reaction and rescreened to enrich for high-affinity binders. This still leaves millions of potential candidate aptamers that need to be sorted into those aptamers with the strongest binding potential. This sorting process usually involves a next-generation (next-gen) sequencing of the pool of aptamers followed by a bioinformatic process to group the “winners” into structural motifs. This challenge of choosing the best candidates from the list of sequences obtained during next-gen sequencing analyses of selected library survivors has been addressed in the recent publication from Ruiz-Ciancio et al.^1^ Previous methods of aptamer analyses using next-gen sequencing have utilized clustering methods based upon seed sequence similarities or folding clusters, both of which require complex sorting of the best sequences for further testing. In the manuscript by Ruiz-Ciancio et al.,^1^ a new method is described that uses computer-based experiential learning to sort through the sequence reads. The resulting program, which they designate Aptamer Runner, provides a tool that takes advantage of the most useful features of existing aptamer-grouping algorithms into a very facile tool for clustering and visualizing aptamer candidates from the enriched library of sequences that bind to a selected ligand. The visualization of clusters of sequence similarities and structural similarities is the major innovation of Aptamer Runner over other aptamer analysis programs. Thus, the final choice of sequences to be further examined is made by the investigator based on the output of Aptamer Runner. Several investigators have already verified the utility of Aptamer Runner.

In the complicated science of aptamer selection, it is refreshing to have a new tool that facilitates the choices of sequences for further testing and use. An additional feature of Aptamer Runner is that, in addition to its predictive features, it searches existing aptamer databases to determine if the selected sequences are related to previously described aptamers. Overall, Aptamer Runner will make SELEX much more user friendly.

## Declaration of interests

The author declares no competing interests.
